# Using a Mixed-Methods Approach to Analyze Traumatic Experiences and Factors of Vulnerability Among Adolescent Victims of Bullying

**DOI:** 10.3389/fpsyt.2019.00890

**Published:** 2020-01-10

**Authors:** Marjorie Roques, Dimitra Laimou, François-David Camps, Anne-Valérie Mazoyer, Mayssa’ El Husseini

**Affiliations:** ^1^LPCN laboratory of psychology of Caen Normandy - EA7452 - MRSH, University of Caen Normandy, Caen, France; ^2^Service de psychiatrie de l’enfant et de l’adolescent, Centre Hospitalier Universitaire de Caen, Caen, France; ^3^Université de Picardie Jules Verne, CHSSC EA 4289, Amiens, France; ^4^Université Paris Descartes, Sorbonne Paris Cité LPCP, Boulogne-Billancourt, France; ^5^Centre de Recherche en Psychopathologie et Psychologie Clinique (C.R.P.P.C.), Université Lumière Lyon 2, Lyon, France; ^6^LCPI laboratoire cliniques pathologique et interculturelle, Université de Toulouse 2, Toulouse, France; ^7^CESP, Faculté de médecine - Université Paris-Sud, Faculté de médecine - UVSQ, INSERM, Université Paris-Saclay, Villejuif, France

**Keywords:** bullying, adolescence, mixed-method approach, projective tests, quantitative tool, clinical interview

## Abstract

A number of studies have analyzed the bullying phenomenon among adolescent victims. Relatively few studies, however, have specifically addressed the associated post-traumatic stress disorder (PTSD). Our clinical practice and therapeutic encounters with adolescents reveal that the majority of bullied adolescents suffer from high levels of PTSD. The objective of this study is to further explore bullied adolescents’ traumatic experiences. In an attempt to analyze these experiences, this article presents a mixed-methods approach. Such an approach will allow to analyze the PTSD that results from bullying as well as subjects’ psychic and family-relevant vulnerabilities. First, bullying will be defined in the context of adolescence. Then the main studies on bullying will be presented. The objectives, tools and methods of analysis will be presented. The interviews will be analyzed according to the Interpretative Phenomenological Analysis (IPA) method. Projective tools, family drawings, Rorschach and Thematic Apperception Test (TAT), will be analyzed using a psychoanalytic interpretation method. Each qualitative tool will be used alongside a validated quantitative tool. The Clinical Administered PTSD Scale (CAPS-CA-5 questionnaire) and the interviews conducted will thus allow to analyze PTSD and traumatic experiences. The Family Assessment Device (FAD) and the family drawing test will enable to assess family functioning; lastly, the Symptom Check List (SCL-90) that will be used alongside Rorschach and TAT tests will allow to analyze individual psychological vulnerabilities. This approach will increase data validity. The originality of this research study is based on a mixed-methods approach, our methodology which is based on clinical psychology, and the choice of certain research tools which have received little attention to date. Ultimately, this study may help improve how bullying is identified and could contribute toward the reinforcement or revision of the criteria that characterize bullying. Lastly, it may help us explore various unexamined dimensions of bullying. A possible limitation is the complexity associated with such a protocol.

## Introduction

Numerous studies have analyzed the issue of bullying among adolescent victims. Relatively few studies, however, have focused specifically on the associated post-traumatic stress disorder (PTSD). Our clinical practice and therapeutic encounters with adolescents reveal that the majority of bullied adolescents suffer from high levels of PTSD. We have thus decided to further explore bullied adolescents’ traumatic experiences. This article presents a mixed-methods approach only recently developed. This approach will allow to analyze the PTSD that results from bullying. It will allow also to better understand subjects’ psychic and family-relevant risk factors. Where family factors are coupled with psychic risk factors, a special convergence (1) may arise, making adolescents more vulnerable to bullying and trauma.

### Defining Bullying

Bullying is a complex global phenomenon (2). Between 100 and 600 million adolescents around the world are supposedly concerned. Over the past 20 years, a considerable number of studies have focused on this phenomenon. Indeed, publications on the issue, written primarily in Spanish and English (3) tripled between 1980 and 2007 (4). Despite clinical psychologists’ and researchers’ keen interest in the subject, the phenomenon remains hard to fathom. Moreover, there is still no consensus on its precise meaning.

Most of the available studies have drawn on Olweus’ (5) definition of school bullying, which is worth quoting at length:

“a negative action when someone intentionally inﬂicts injury or discomfort upon another, basically what is implied in the deﬁnition of aggressive behavior. Negative actions can be carried out by physical contact, by words, or in other ways, such as making faces or mean gestures, and intentional exclusion from a group. In order to use the term bullying, there should also be an imbalance in strength (an asymmetric power relationship): the student who is exposed to the negative actions has diﬃculty defending him-/herself and is somewhat helpless against the student or students who harass. (…) The phenomenon of bullying is thus characterized by the following criteria: it is aggressive behavior or intentional ‛harm doing’, which is carried out repeatedly and over time in an interpersonal relationship characterized by an imbalance of power” (5, pp. 8–9).

This definition, which covers most of the important characteristics of bullying, seems fairly comprehensive. Thus the Olweus’ definition will be chosen for the present study.

Some authors have described different types of bullying: direct, indirect and cyber bullying (6; see Table p. 880).

1. Direct bullying. This type of bullying is more common among boys. It includes physical and verbal attacks.
2. Indirect bullying. This type of bullying is more common among girls and more hidden. It includes gossiping, spreading rumors, social exclusion or even the deterioration of the victim’s objects.
3. Cyber bullying. For Patchin and Hinduja (7), cyber bullying is voluntary and repeated harm inflicted through the use of technologies. Using technologies, bullies can extend the reach of their intimidation and their grip beyond the “usual” space where bullying takes place (often in school). This type of harassment includes obscene or insulting messages sent by telephone or computers, rumors spread on the internet or even sites created specifically to humiliate the person (use or dissemination of compromising photos or videos). Because of the anonymity promoted by technologies, these bullies have some form of power.

In addition to the elements of the Olweus’ (8) definition (frequency, intensity, repetition, duration, power of imbalance), the specificities of adolescence should be considered. In a previous work (9) two essential dimensions have been highlighted, i.e., the psychic processes associated with puberty, and the significant role played by the peer group.

To develop our methodology, we will focus on these two dimensions which we deemed relevant.

### Adolescence and Bullying

According to Volk et al. (10), bullying is at its “maximum level” when it occurs in adolescence. Adolescence is a particularly sensitive period. It is conductive to the reactivation of the vulnerabilities experienced in childhood and the emergence of anxieties, mainly because of sexual maturation (11–15). Some authors speak of a specific adaptive strategy during this period (16–18) which allows the development of romantic relationships (19) and enables some adolescents to distance themselves from sexuality (20). Sometimes, sexuality during adolescence induces anxiety. Moreover, the quest for autonomy in adolescence requires a sense of internal security. This enables them to cope with separation and its accompanying emotions. Nevertheless, the separation process may destabilize the already precarious equilibrium of some vulnerable adolescents (9).

The significant role played by peer groups in adolescence sheds further light on bullying (21, 22). Renshaw, Roberson and Hammons (23) analysed the roles different protagonists play: “ringleaders”, who start the bullying; “followers”, i.e. those who participate in the bullying; “supporters”, who encourage the bully or make fun of the victim; “defenders”, who come to the help of the victim; “witnesses”, who passively watch without intervening, especially in the school setting (24, 25); “victims”, and lastly, the “neutral”.

Indeed, peer groups are perceived as providing support (26, 27) and play a major role in adolescents’ identity construction (28). The group may represent a secure space and provide the narcissistic value associated with adolescence (29). Nevertheless, it may also be a source of danger for the most vulnerable adolescents. The group acts as the guarantor of the very existence of some adolescents (30) as it is perceived as a space for protection, valuation, and socialisation. A number of studies have repeatedly highlighted the paradox of the peer group. As a matter of fact, the group can be used as a weapon in the service of bullying but also as a resource to allow the young to overcome bullying (31–33).

Anzieu and Martin (34) call attention to the fact that the group may have a deleterious effect on the personality of a member, in terms of his or her integrity, freedom and independence. The group is formed through the fusion of its members. Through this process a collective identity is created. It is often led by a leader who guides and secures its members who risk losing all critical and personal judgment. Regularly, a scapegoat (35) is designated based on his or her ‘scapegoat features’, i.e., any differences from the group (infirmity, physical or social difference, disability, ethnic origin) as well as ‘extreme’ qualities (wealth or poverty, beauty or ugliness, vice or virtue, strength or weakness) which awaken hatred, desire, or covetousness (36). It is thus easy to understand, in the context of bullying, the extent to which the group has a significant impact on subjects (37). Indeed, for adolescents, the peer group and the group processes, supports the identity construction.

### Family and Psychological Risk Factors in Adolescent Victims

It is worth mentioning that these two aspects alone (adolescence and the peer group) do not explain the phenomenon. Other factors must be taken into account. Other possible dimensions will thus be highlighted in order to better understand why some adolescents are targeted. Most authors agree that victims of bullying do not fall within a specific personality or profile (38). Nevertheless, specific adolescents are targeted, as bullying depends on certain factors that may increase the emergence of this process and encourage its expression and establishment.

Relatively few studies have focused on family risk factors. These studies underscore that the economic and social conditions of the family (39), intra-family violence (40), intrafamilial sexual abuse (41) and family equilibrium disruption may precede bullying (42). Moreover, the mental health of parents (43, 44), parenting styles, and the quality of the attachment between adolescents and their parents (42,) have also been identified as risk factors (46). Overprotection and poor identification with parents affects the degree of victimization by peers (5, 47, 48). For Finnegan et al. (49) victimization is associated with perceived maternal overprotection for boys and with perceived maternal rejection for girls. Victimization has been also associated with greater parental involvement in school, which may reflect parental awareness of children’s difficulties but which may also reflect a reduced independence among these youths (50, 51). In a recent study (52), the researchers demonstrate that the parental bonding quality (care, indifference, overprotection and encouragement of autonomy) is related with children’s bullying/victimization experiences and post-traumatic symptomatology.

Despite the significance of the factors cited above, the general consensus among researchers is that individual psychological risk factors play a great role. More studies have found that bullied youth have more psychotic personality (53, 54) or display more neurotic traits (i.e., they suffer from emotional instability) (55–56) compared to the general population. Moreover they are more extroverts (45).

A recent randomized study conducted in Greece (52) sought a statistical analysis of the relationship between the symptomatology of PTSD and the role of parental bonding in the victimization process (as a risk factor and as a protection factor). The researchers used the the Greek version of the revised Olweus Bully/victim Questionnaire and the Children’s Report of post traumatic symptoms. They examined how traumatic symptom levels (depression, somatization, avoidance behavior, dissociation) are associated with parental bonding type, in the context of bullying type, and how bullying behavior roles are shaped. They analyzed a specific model that emphasizes how certain types of parental bonding can cause certain emotional reactions in relation to bullying and traumatic symptoms.

### Bullying and Negative Psychological Effects in Adolescent Victims

Exposure to bullying is a significant risk factor that contributes independently to the emergence of psychological difficulties and pathology, regardless of pre-existing mental health symptomatology, genetic predisposition, or family psychosocial difficulties (57).

According to Bhui et al. (58) adolescents who have been victims of bullying show signs of significant psychological distress and social integration difficulties. They also suffer from more psychosomatic disorders (59) and more psychopathologies than the normal population (60). The victims present also other psychological difficulties (61), such as sleep disorders (62), depressive symptoms (27), and anxiety (the most common is social phobia (63). In addition to this, they are much more prone to self-harm (Mahon Mc et al., (64, 65); suicidal ideation, or suicide attempts (67).

They also have symptoms of post-traumatic stress resulting from the bullying (9, 67–70). Kaess (68) argues that bullying should be viewed as any other form of violence. This view is shared by other studies (67, 69) which have found a high level of PTSD in victims using the Life Event Checklist. Moreover, the studies have found that in some cases the consequences of adolescent bullying are more serious than those faced by victims of serious abuse or neglect (68, 71). In a previous qualitative study (9) the symptoms of trauma (negative effects of trauma) that are still present even one-and-a-half years after the bullying event, have been identified. In addition to this, drawing on a case study (Alexandra, 16 years old) and using interviews and thematic analysis, the adolescent’s subjective traumatic experience could be explored.

## Research Objectives

Following our literature review on bullying and drawing on our clinical practice, two main objectives were defined.

The first objective is to explore the family and individual risk factors associated with adolescent victims of bullying.

The second objective is to explore the PTSD symptoms linked to bullying.

In order to explore the above mentioned dimensions, a clinical assessment protocol combining qualitative and quantitative tools will be applied.

## Methodological and Epistemological Considerations

Given the predominance of quantitative studies, a purely qualitative approach may have been privileged as in the study undertaken by (72) (one of the few studies that have used this approach to analyze bullying). However, like Guerra et al. (73), we believe that combining quantitative and qualitative approaches may make it possible to benefit from the richness of the qualitative method while maintaining the rigor of the quantitative method. Working in the field of suicidology, (74) have described several advantages of using both quantitative and qualitative approaches. They argue that qualitative studies can help us to interpret and understand the relationships between variables used in quantitative studies (75). In addition to this, given that a mixed-methods approach associates a multitude of elements, the results will potentiated and enhanced. In their article, they present three possible outcomes using quantitative and qualitative approaches in an integrated way, with which we agree: “1) the results are complementary and thus provide a fuller picture of how things are related to each other (…); the results are convergent and thus contribute to validate each other; or (3) the results are contradictory and then more research is needed (75).” (p. 78).

With regard to qualitative methods, in order to understand the phenomenon, the phenomenological approach, which favors an inductive approach to access the psychic reality of subjects will be privileged. In this approach, “the researcher dwells on a specific meaning, which may be a moment, a sentence, an omission or a syntactic element” (76, p. 181). The psychoanalytic approach, which integrates both inductive and deductive approaches, will also be used.

Although distinct from the phenomenological approach, the psychoanalytic approach nevertheless proposes a holistic approach to the subject. The subject and his/her experience are thus perceived as the objects of research. Analyzing a subject’s experience requires “(…) as the sole instrument of measure the observer’s relationship to the observed, the observer’s relationship to his/her observation, and the space in which these relationships unfold, which are as much the effect as the instrument of this “measure” (77, p. 177). Numerous quantitative methods do not place the same importance on the relationship between a subject and a researcher and on subjective factors (78). Unlike these studies, the analysis of the impact of relational and subjective factors is at the heart of a qualitative research approach.

Moreover, both the phenomenological and psychoanalytic approach support a hermeneutic approach. They are therefore convergent insofar as they place emphasis on the subjectivity of the researcher and on his/her interpretation of situations. In addition, they both require constant back-and-forth communication between the material and how it is interpreted. This approach encourages the emergence of original data and hypotheses (79). Both approaches allow the formulation of hypotheses that are not simply explanatory proposals but are rather theoretical and clinical constructs. These hypotheses link theory and practice based on what the researcher observes and interprets.

With regard to quantitative methods, a conventional approach that uses validated tools is privileged.

## Characteristics of the Research Population and the Formation of Subgroups

The study will include a broad sample of school-going adolescents, girls and boys, aged between 12 and 18 whose bullying had stopped for at least a month. This decision will make it possible to evaluate the symptoms of PTSD when the bullying incident had occurred in the past, based on the international classification criteria (DSM V). Given that our protocol includes both a quantitative and a qualitative section, only a small number of patients can be included in the study. Approximately 60 patients will participate in the study.

We set up subgroups based on the following variables:

Age (a group of “younger” adolescents (12–15 years) and a group of “older” adolescents (15–18 years). According to the available literature, the problems encountered differ depending on adolescents’ age;
The duration of the bullying, which would eventually determine the intensity of the trauma;
Gender. Studies on bullying have regularly brought up the issue of gender (80, 81): boys (from 8.6% to 45.2%) are more affected than girls (from 4.8% to 35.8%) (82).
The type of bullying; all types of bullying described in the literature will be studied: direct bulling (psychical and verbal attacks), indirect bullying (e.g. gossiping, rumors and social exclusion) and cyber bulling (bulling through technologies). The whole clinical protocol will be applied to adolescents who have suffered the above mentioned types of bulling.

Certain non-inclusion criteria will be applied. The objective of our research is not to study the acute phase (stress), but rather to explore the so-called “post-traumatic” phase. Subjects who are currently victims of bullying are thus excluded from the study. The family-relevant and psychological risk factors that will be identified will be analyzed using tools (interviews, questionnaires, Rorschach, TAT and family drawings) that assess multiple variables.

## Research Tools

In this section, further details will be provided about the tools used by categorizing them according to their characteristics (qualitative, quantitative):

### Qualitative Tools

#### Interviews

Data will be collected *via* semi-structured interviews. The semi-structured interview is a method that combine an approximate standardization of questions with the possibility for the participants to develop and detail their answers when needed. The authors reviewed the international and national literature on bullying to develop a guide for these interviews. Every interview will be audio-taped for later transcription, with the participants’ permission, and transcribed verbatim in French.

Open-ended questions will target broad topics related to our research area of investigation while allowing participants to interpret them subjectively and narrate their personal experience of the bullying. The topics covered include relations with family and peers and bullying and trauma experiences.

In relation to the two research objectives mentioned above (cf. research objectives), i.e., the study of individual family and psychological risk factors (objective 1) and the evaluation of the subjective trauma dimension (objective 2), relationships with others will be explored based on the following question: “Could you tell me about yourself and about your family and friends?” (interview 1-> objective 1). The subjective trauma dimension will also be analyzed based on the following question: “Can you tell me about the bullying you were subjected to?” (interview 2 -> objective 2).

Participants can choose the interview site. Their narrative determines the length of the interview. Two different researchers will conduct these interviews, separately. Each had training in the fields of bullying and qualitative research methods. Interviewers will use prompts and probes as needed to encourage the participants to develop their narrative and give a detailed account.

#### Data Analysis

Aiming to inquire about a clear representation of the participants’ experience of bullying and its impact on their psychological functioning, the authors have resorted to a phenomenological research design for the qualitative interview. Phenomenology is a nonprescriptive approach to research that allows the essence of experience to emerge, while anchoring data analysis in the participants’ unique representations (83). The aim is to explore personal experience and the subjective perception of a phenomenon. Our research approach is phenomenological in that it involves detailed examination of the participants’ personal perceptions and lived experiences.

In this perspective, the Interpretative Phenomenological Analysis (IPA) will be used to analyze the interview data. The IPA, developed by Smith in 1996 in the field of health psychology (84), is an established qualitative methodology used to explore in depth how individuals perceive particular situations they are facing and how they make sense of their personal and social world (85, 86).

Built upon the principles of the IPA, an in-depth qualitative analysis will be conducted, starting with detailed case-by-case study of each interview transcript, according to an iterative inductive process. Researchers will proceed with several close detailed readings of each interview to provide a holistic perspective, noting points of interest and significance. They will then proceed, following a step by step analysis, to the description of the analytic themes and their interconnections respecting the link back to the original account of the participants. The analysis navigates between different levels of interpretation leading, at a last stage, to the production of a coherent ordered table of the emergent themes. This procedure is inductive and the analysis of the literature data is done in a later stage. The size of the sample is not decided beforehand but determined by data saturation: once the in depth analysis of the interviews doesn’t provide any new themes. Following this process, the researchers can redefine the research question, look for counter-examples and hence investigate new pathways.

For validity purposes, the researches compare their coding. Two trained researchers proceed to the coding independently then discuss the emerging codes with two other research members who had read the transcripts. Independent verification aims to clarify the identified codes and ensure they accurately reflect the data collected. To increase the validity of the data, the subjectivity of one of the two researchers currently analyzing the verbatim report of the semi-structured interviews conducted among adolescents will be compared with that of the other^1^. These interviews were undertaken by different researchers who were not involved in the analysis process. This is in an attempt to reduce interpretations made following projections or the manifestation of transference. IPA-trained researchers in charge of the analysis will select common identified themes and formulate interpretive hypotheses. They will work under the supervision of an IPA-trained researcher and a researcher trained in psychoanalysis.

#### Projective Tests

Projective tests will complete the analysis of the verbatim reports of the interviews by promoting “the discovery of manifestations possibly too discreet to be grasped (…) by an analytical ear” (83, p. 1). They will allow us to assess unconscious psychic processes and to identify the vulnerabilities, resources and frameworks of functioning a subject maintains; this is something interviews are unable to do. Indeed, a small number of interviews alone cannot help identity a subject’s unconscious thoughts because the study will be conducted a limited in period.

In clinical research, projective material makes it possible, over a very short period of time, to assess psychic processes otherwise difficult to access. It provides a snapshot of the unconscious issues that underlie the subject’s psychic organization, the manifest cover of symptoms, and the discourse of patients Chabert (87). By this we refer to the possibility of accessing latent content based on the analysis of the manifest responses and narratives provided by the patient during the presentation of symbolic cards. The use of projective tests offers researchers in psychopathology and psychoanalysis the opportunity to test their hypotheses across a broad section of patients. If a researcher observes major differences and/or similarities in a significant number of subjects, he/she can then identify general trends (88–90). It must be said, however, that this generalization of results occurs without overlooking the case study and psychoanalysis.

The Rorschach and TAT (Thematic Apperception Test) projective tests will be used in this study, according to the method developed by the School of Paris. The School of Paris has attempted to show the value of this test, using an approach that draws on the psychoanalytic theory of psychic or psychopathological functioning. The School of Paris (or the French school) comprises a group of university researchers who use the Rorschach and TAT methods of analysis, which are both quantitative and qualitative (91). They draw from the studies undertaken by Anzieu (88), Rausch de Traubenberg (92) and Chabert (93–96). From this perspective, Rorschach and TAT are not perceived as “tests”, as they do not meet the psychometric conditions of a real test (87), but as “clinical assessments”. A projective test is thus capable of revealing defensive mechanisms and psychic conflicts inherent in the psychic functioning of a given subject. Projective tests are therefore not intended to differentiate between individuals based on a specific factor, but to distinguish between the modalities of psychic functioning, *or intrapsychic qualities*, and to evaluate their psychic importance in order to describe the personality of the subject. A projective test allows for changes in personality to be identified depending on internal or external stimuli (such as maternity, menopause or trauma). These tests can thus be used to measure the degree to which an event influences an individual’s personality (92), as in the case of bullying. Our past studies identified trauma-related indicators relating to the Rorschach test in the field of sexual violence in adolescents (97,) and to the TAT test in the field of adults with serious illnesses (99). These indicators are present irrespective of subjects’ psychic functioning. Projective tests are thus capable of differentiating what belongs to the present, and therefore to the traumatic register, from what belongs to normal psychic organization (100).

The Rorschach test is a clinical psychological assessment developed by H. Rorschach in 1921 (101). The test consists of a series of 10 ink blots and is a projective psychological test: through the responses they give, subjects freely project the elements of their internal world, their conflicts, their fantasies and their defense mechanisms, based on the material presented. While some boards are polychromatic, others are monochromatic. Rorschach is a free-response test which uses non-representational images. It is widely used in clinical psychology (102). While this test can be interpreted in widely varying ways, two major interpretations predominate today: the integrated Exnerien system (103) in English-speaking countries and the psychoanalytic approach of the School of Paris in Latin countries. The validity and robustness of the Comprehensive System approach to Rorschach interpretation has been established by numerous statistical studies (104) and by several meta-analyses (105) in comparison with MMPI (106, 107). The analysis recommended by the School of Paris relies on both quantitative elements based on normative data (108): number of responses, quality of responses etc. and qualitative elements: perception of latent content, expression of affects, etc. Diagnosis is based on a comprehensive and psychodynamic approach to these factors. The stability of the Rorschach test was highlighted by the studies undertaken by Piotrowski and Schreiber (109).

The T.A.T. (Thematic Apperception Test) is a projective test used together with the Rorschach according to the recommendations of the School of Paris (110). It consists of a series of cards depicting figurative drawings, photos and reproductions of engravings, in black and white, and more abstract drawings. Subjects are asked to invent a story for each card. The test was developed by Murray in 1935 (111). In the 1950s in France, Shentoub proposed a new method to analyze TAT cards based on the analysis of the narratives provided using a discourse analysis grid (112, 113) to reveal subjects’ unconscious thoughts and, above all, their defensive mechanisms.

In this field, as in the field of psychoanalytic therapy with patients, regardless of subjects’ age and the context of the encounter, the analysis and interpretation of responses leads to the formulation of clinical hypotheses^2^, underpinned by the identification of several facets of psychic life:

Defensive strategies,
Anxieties,
Impulsiveness,
The expression of emotions,
The capacity for mentalization (114, 115).

The defensive mechanisms in the Rorschach are observed, among other things, thanks to:

The modes of apprehension of the material their successions within each board and within the protocol;
The quality of the answers and their contents.

At the TAT, it is the score sheet (113) that will make it possible to specify the subject’s defensive register.

Anxieties in Rorschach, can be observed by studying the quality of the so-called “Clear Obscure” responses (Clob, FClob, ClobF). In the TAT, anxiety is expressed directly in the content of the stories and in the themes of the latter as well as in the impact it has on the processes of secondarization through the processes of discourse marking inhibition (CI) and discourse alteration (E4).

Impulsiveness in Rorschach is observed through factors such as:

abstract responses:
formal determinants of poor quality;
pure color responses;
overall responses of poor quality;
perseverations;
a special attention will be paid to the red color and red cards.

In the TAT, impulsiveness is expressed in the use of behaviors that can be observed during the passing of the test or through the quality of the stories.

The expression of emotions in Rorschach can be observed, among other things, through the Type of Intimate Resonance (T.R.I.) and the Complementary Formula of quantitative data, but also the formal quality of responses taking into account color or achromatism. In T.A.T., emotions are observed trough the stories and referred to in the different series of the score sheet (113).

Mentalization capacities in Rorschach are observed, among other things, through overall productivity, the large number of kinesthetic responses, in particular human kinesthetics, formal indicators no higher than the norm for F% and F+% and even slightly lower than the norm for F%, the percentage of animal responses (A%), the presence of global responses associated with human kinesthetics and a “sensitivity” to the latent symbolic of the plates (116). In the TAT, it is the productivity, dramatization and secondarization of stories that will sign the capacities of mentalization (112).

These factors are not exhaustive.

Applying a mixed-methods approach to these complex data according to the precepts of the School of Paris can allow us to restore the psychic dynamics underlying observable symptomatic manifestations and to question their place within the subject’s psychic economy.

Using both Rorschach and TAT tests is likely to give rise to data discrepancies, with a discontinuity in the results possibly interpreted as a result (117–119). Generally speaking, the interdependence of the two tests provides access to more comprehensive and diverse information that can enrich the study.

The scoring of projective tests is based on a manual that was developed following the statistical analysis of a large sample (108) or on a discourse analysis sheet (113). To ensure the greatest consistency possible across certain responses, the Thematic Apperception Test (TAT) (120) proposes an interpretation of discourse procedures and is complementary to the phenomenological method applied to interviews. Thus, in addition to the IPA and its effectiveness, projective tests can allow us to explore other aspects of patients’ psychic reality and to complete or corroborate the information obtained through interviews and the SCL-90 regarding the psychological profile.

#### The Family Drawing

In order to assess families’ risk factors (hypothesis 2), the family drawing test (121, 122) will be privileged. Unlike in classical projective tests (Rorschach and TAT), in which perceptual material is imposed, the advantage of drawings is that they incorporate an additional dimension, i.e., the adolescent’s creativity developed on the basis of his/her own subjectivity. Although drawings have long been used for diagnostic purposes in clinical practice (123), their reliability and validity has never been clearly proven. Handler and Habenicht (124) underscore the need for normative data and argue that the interpretive approach of the clinical psychologist who uses this type of tool must be analyzed^3^. Particular caution must be taken when using this tool insofar as it can only have meaning in relation to other tools (126). Specifically, in the case of the present study this refers to the FAD questionnaire and interviews.

In psychoanalysis, Klein, A. Freud and Winnicott revealed that drawings were equivalent to dreams. Drawing is one of the preferred modes of communication of the child and is favored for initiating communication with children in psychotherapy. But what about drawing in adolescence? Several authors believe that adolescents’ drawings have no clinical value because at this age young people have reached the stage of visual realism. For Corman (121), however, although drawings challenge adolescents’ Ego ideal and reveal the self-imposed challenges in play, their drawings nevertheless reveal their capacity for regression and may provide access to the manifestations of the unconscious. While adolescents may be trapped within a certain perceptual truth that may limit their imagination, projection remains predominant. Birraux (127) refers to projection as a “tool for adolescence” and underscores the role it plays in regulating impulses specific to adolescence.

The choice of family drawings with teenagers was motivated by our clinical practice with this population. Indeed, this practice has shown that some young people are not prone to verbalization or do not have the necessary hindsight to express their experiences. Paradoxically, those who engage in verbose discussions may be reluctant to talk about their family environment during interviews as it is a subject that is particularly sensitive in this period of life. Moreover, adolescents are often reluctant to respond to questionnaires or interviews, and drawings thus allow access to their representations of their families. Used as a mediating tool7, family drawings are thus the least direct method through which to access adolescents’ representations of their families (128). Using this medium, adolescents can express their experiences, feelings and desires in relation to their family history. According to Gross and Hayne (129), drawings facilitate language development and promote the expression of emotions. Drew et al. (130) advocate the use of photography as a medium that encourages adolescents’ involvement in research and enhances qualitative data. It is worth mentioning, however, that this does not imply that adolescents are “made” to say what they do not want to say; rather, they are helped to express what they are unable to express clearly. In this sense, analyzing adolescents’ drawings allows access to underlying family issues and the meaning these young people attribute to them. Put differently, the “quality” of the drawing and the skills involved in the production of a drawing are of no importance; what is analyzed is the level of development according to the adolescents’ age.

In terms of material, subjects will be given an A4-sized paper, positioned horizontally. Pencils and colored pencils will be presented in a way that will enable them to decide freely whether to use them or not. The following instructions will be given: “Draw your family”, after which they will be asked to draw *a* family. They will be thus required to produce two drawings.

To analyze the drawings, the researcher will focus on:

The order in which items will be drawn
The logical sequence (or absence of sequence) in which the characters will be drawn (head, body, arms, color, etc.)
How the characters will be situated in the available space (where the drawing will be situated on the paper, whether characters will be drawn at greater or lesser distance from each other, etc.);
Complete drawings (or not) of the characters and their emotional expressions;
Whether the gestures, mimicking and verbalizations of the subject will accord with the drawing made.

The rating and analysis will draw on an amended version of Corman’s methodology (121), revised by Jourdan-Ionescu and Lachance (122) within a psychoanalytic framework. Redundant characteristics, i.e., unhabitual characteristics, will then be removed in order to highlight the general trends. The book “Family drawing” (122) contains all the elements necessary for rating and interpretation. It is something of a handbook for clinical psychology researchers.

**Figure 1:**
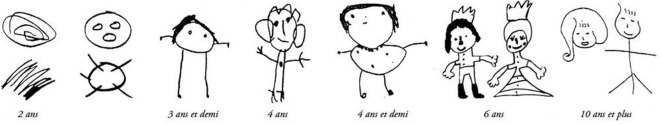
Drawings corresponding to each developmental stage.

Several important elements must be taken into account when analyzing a family drawing (122). These include:

Mentalization, as defined by Fonagy (115), in relation to the developmental level revealed by the drawing. **Figure 1** illustrates drawings with key characteristics of the different developmental stages of children.
Any other “usual” element should be highlighted. For example, there are minor (slight variations in form) or major (scotomas) stereotypical forms that may reveal certain intra-family vulnerabilities (for example, the hand linking an adolescent to his mother may be missing). Or there may be an absence of facial features, which according to Burns (131), refers to inadequate contact with one’s environment.
By comparing the two drawings or analyzing an adolescent’s potential refusal to draw “a” family, the different types of identification are revealed. Corman (121) has described the identifications of *reality* (the subject is realistically drawn), desire (the projection of tendencies onto another character: in teenagers, this is often the baby who shows nostalgia for an ideal age) and *defense* to avoid guilt by identifying with the person perceived as punitive.
The representation of self in the family group through the order of the characters drawn and the care with which the different characters will be drawn. According to Van Krevelen (132) the father is the first character drawn, then the mother (in larger proportions), then the children who are drawn in chronological order according to their age. Forgetting to draw oneself may reveal a fragile representation of self or devaluation.
The nature of family ties revealed by how the different family members are positioned, the roles of each family member, and the distance (more or less) between family members;
Issues concerning the attachment and separation of the family group: are the hands and feet drawn? Can the adolescent imagine a family other than his/her own? Is the drawing colored? For example, coloring of parents and/or children and the adolescents themselves. According to Widlocher (133), the color black takes on a particular significance at adolescence and reveals modesty and restraint with regard to feelings. The dominance of black and white areas may refer to a certain depressivity, inhibition, or a split-off affect state (134).

### Quantitative Tools

After presenting our qualitative tools, below are the quantitative tools employed in the mixed-methods approach. We chose to associate a qualitative tool with a quantitative tool in order to improve data validity.

#### The Family Assessment Device (FAD) Questionnaire

The FAD is a self-report questionnaire that assesses family functioning. It is filled in by only one member of the family. This tool was developed to evaluate the different dimensions of the McMaster Model of Family Functioning (135, 136). One sub-section of this tool (the general functioning) had been validated in its French version. This is the only sub section whose internal cohesion has been proven to be strongly correlated to all the other subscales (137). The general functioning subscale, which comprises 60 items, and a Likert scale will be used. The FAD proposes a number of statements about families (such as, for instance: When someone is upset the others know why). Participants are asked to read each statement carefully and to choose the one that best describes their family. Depending on their perception of their families, they must then give their response on a Likert scale ranging from “Strongly agree” to “Strongly disagree”.

#### The CAPS-CA-5 Questionnaire

To assess PTSD and to respond to the first research objectives, several tools were selected. Given that the objective are to explore both the traumatic experience and the impact of adolescent bullying, the Clinical Administered PTSD Scale (CAPS-CA-5) for children over 7 years old (138) will be used. This tool seems to be the best suited for our purposes. The CAPS-5 is a 45–60 minute semi-structured interview that can be administered by clinical psychologists and researchers and has 30 items. The tool allows therapists to:

establish the current diagnosis (in the last month) of PTSD;
make a prognosis of the evolution of PTSD;
assess the symptoms of PTSD over the past week.

The CAPS-5 complements the semiology of PTSD based on the Diagnostic and Statistical Manual of Mental Disorders—Fifth Edition (DSM-V) criteria with questions that make it possible to explore—in this study—the different criteria for bullying. Naturally, the questionnaire does not specifically focus on bullying but it is rather appropriate for this study. The questions revolve around:

the onset, duration, intensity, severity and frequency of symptoms (With the CAPS 5, the clinical psychologist combines information relating to frequency and intensity to create a unique severity score);
subjective distress;
the impact of symptoms on social functioning.

For each symptom, standardized questions are provided. The administration of the scale requires the identification of a single index trauma to serve as the basis of symptom inquiry. Other than the Criterion A inquiry included in the CAPS-5, the Life Events Checklist for DSM-5 (LEC-5) is recommended.

The original version of the CAPS has a very high psychometric quality (139). A French–Canadian version of the CAPS is available (140).

#### The SCL-90 Questionnaire (Symptom Check List)

The SCL-90 (141) has been validated with adolescents. An American and a French study (141, 142) revealed a satisfactory internal structure and convergence validity for the nine sub-sections. The SCL-90 assesses psychological distress and adolescents’ psychopathological profiles using items that represent nine symptom dimensions. It consists of 90 items. According to Derogatis, “SCL-90 is a self-report inventory of symptoms designed to reflect the psychological profiles of “normal” populations or individuals with organic or psychiatric pathologies” (ibid. p.5). Moreover, SCL-90-R is a measure of the current state, at this moment in time, of psychological symptomatology. It is not a direct measure of personality. This questionnaire is rated and interpreted according to nine symptomatic dimensions (somatization, obsession–compulsion, interpersonal sensitivity, depression, anxiety, hostility, phobic anxiety, paranoid ideation, and psychoticism) and three global indices of distress (Global Severity Index, Positive Symptom Distress Index (intensity), and the Positive Symptom Total (number of symptoms). This tool consists of a list of problems that people sometimes encounter. Respondents are asked to mark one numbered circle reflecting the extent to which the problems on the checklist have bothered them over the last seven days, including at the moment of completing the questionnaire [(0) not at all, (1) a little bit, (2) moderately, (3) quite a bit, and (4) extremely]. The responses are scored on a 5-point scale ranging from “not at all” to “extremely.” The nine symptomatic dimensions are listed below:

Somatization (SOM)
Obsession–Compulsion (O–C)
interpersonal sensitivity (SENS)
Depression (DEP)
Anxiety (ANX)
Hostility (HOS)
Phobic anxiety (PHOB)
Paranoid ideation (PAR)
Psychoticism (PSY)
+ ADDITIONAL ITEMS (various symptoms)

Combining data from the CAPS-CA-5 and SCL-90 questionnaires could be advantageous insofar as it may lead to the emergence of correlations between the symptom profile, symptoms of PTSD and the level of psychological distress. While it is difficult to know what to expect, comparing these two questionnaires may put into perspective certain psychological tendencies among these young people.

Each tool will evaluate a different dimension (psychological, family or bullying experiences), corresponding to each of our two hypotheses. The tools will first be treated separately before comparing the data obtained in line with our hypotheses (for instance: The FAD and family drawing test to analyze families’ vulnerabilities). Lastly, all the data obtained using the different tools will be compared in order to propose a comprehensive analysis of the phenomenon.

**Figure 2:**
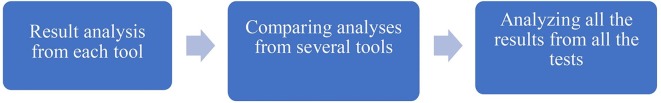
Steps of each tool analysis.

These different tools will evaluate common dimensions or concepts, thereby increasing the validity of the data and the comprehensiveness of the analysis. For example, mentalization will be assessed using four tools (Rorschach, TAT, family drawing, and interviews), or the trauma will be assessed using the CAPS-CA-5, the SCL-90 and the interviews. Each concept will be studied in relation to the specificity of each tool and then, compared through the tools, which will broaden its understanding. **Figure 2** illustrates the different steps of each tool analysis.

## Location Where Data Collection Will Take Place

To recruit participants, public healthcare institutions and schools in which adolescents may be identified who meet our inclusion criteria will be contacted. The research will take place in France. Big cities of France will be targeted (e.g. Paris, Lyon).

## Data Collection Methods

### Before the Study

An informative letter will initially be sent to the director of each institution to inform him/her of the study as well as the methodological procedures. In the letter, we will request him/her to put us into contact with adolescent victims of bullying. To participate in the study, both the adolescents and their parents will be expected to provide their written, informed consent. In addition, official approval will also be obtained from the institutions before meeting the subjects (ethical review board). Once the various consents have been obtained, information meetings will be held with the adolescents’ reference persons to inform them about the study’s inclusion criteria and to respond to their questions if necessary.

For ethical reasons, and given that this is a sensitive sector of the population, it will be ensured that these adolescents are already receiving help (psychiatrically or psychologically) or that will be receiving such help once the research ends. Interviews will be carried out at less sensitive times for the adolescents involved (for example, they will not be conducted after a serious bullying episode).

**Figure 3:**
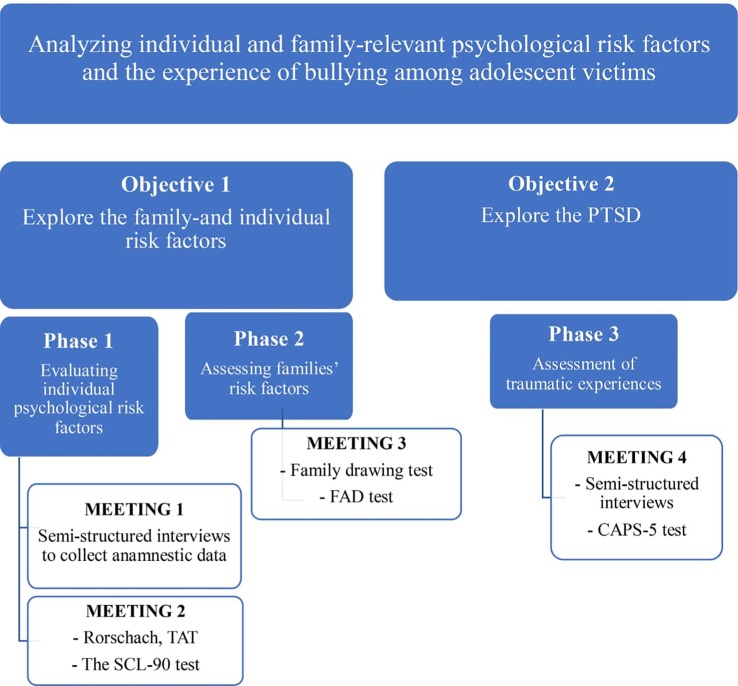
Objectives, phases and tools.

We will then conduct the study following the steps and using the tools presented in **Figure 3**.

## Limitations, Strengths and Perspectives

Our objective was to submit a proposal involving a mixed-methods approach to be appraised by the scientific community in order to undertake a study among/of adolescent victims of bullying. This methodological reflection emerges from our experience gained through clinical observations of this population. We consider that this is a positive point of this work. Experimenting with this methodology and using tools habitually used in clinical settings, may enrich qualitative research.

The proposed tools have already been tested in routine care settings. But what may be expected within the context of a research study? The implementation of this kind of approach is maybe the most original point of this research. As stated by Plexousakis, et al. (52), who have made a research on bullying through quantitative tools: “future research should also include qualitative methods (interviews, etc.) that engage bullies and victims so as to clarify a deeper understanding of bullying and parental attitude or family relational dynamics through children’s and adolescents’ personal narrative/experience and a discourse analysis methodology…” (52)’’. We strongly believe that the introduction of qualitative tools will contribute to explore various unexamined dimensions of bullying, to improve treatment and prevention and to create different research protocols. The mixed-methods approach that will be implemented will provide insights which may help improve how bullying is identified. The findings could help reinforce or revise the criteria that characterize bullying. Finally, thanks to this mixed design, this methodology may prove to have a solid scientific foundation. Therefore, the main objective is to link research with clinical practice based on the psychoanalytic field.

A more “clinical” benefit would be the ability to identify suffering adolescents. Due to the interest that the concerned adolescents will receive, along with their feeling that they are being listened to, this research design may lead to the establishment of an alliance. Those not receiving counselling can thus be encouraged to integrate a specific care program or can be guided towards a more singular and individual therapy corresponding with the research. Lastly, the tools tested using this method could be used to assess its effectiveness.

A possible limitation will be the complexity associated with such a protocol. This is indeed a time-consuming and complex protocol because of the number of tools involved and it could lead to a loss of participants. Another negative point of the approach presented in this paper is linked to the fact that it may be difficult to know if the individual characteristics that will be detected through this clinical protocol, pre-existed before the trauma of bullying or whether they are the result of bullying. We believe that the use of a qualitative approach will allow to better distinguish between the latter timing.

## Author Contributions

MR and DL are equal contributors to this paper (co-first authors). They both contributed to writing the paper. They have co-designed the method proposed. They have co-constructed the different parts of this paper. A-VM, F-DC and MH contributed to the choice of the tools that will be used and to writing some parts of the paper. All authors read and approved the final manuscript.

## Conflict of Interest

The authors declare that the research was conducted in the absence of any commercial or financial relationships that could be construed as a potential conflict of interest.

## Footnotes

1 Several subjectivities will be a guarantee of a certain scientific "objectivity."

2 In this, it is akin to the inductive approach that the grounded theory approach values.

3 They focus here on “The kinetic family drawing technique” (125)
